# The Plebeian Algorithm: A Democratic Approach to Censorship and Moderation

**DOI:** 10.2196/32427

**Published:** 2021-12-21

**Authors:** Benjamin Fedoruk, Harrison Nelson, Russell Frost, Kai Fucile Ladouceur

**Affiliations:** 1 Faculty of Science University of Ontario Institute of Technology Oshawa, ON Canada; 2 Faculty of Health Sciences Queen's University Kingston, ON Canada; 3 Faculty of Engineering Lakehead University Thunder Bay, ON Canada; 4 School of Engineering Technology, Trades, and Aviation Confederation College Thunder Bay, ON Canada

**Keywords:** infodemiology, misinformation, algorithm, social media, plebeian, natural language processing, sentiment analysis, sentiment, trust, decision-making, COVID-19

## Abstract

**Background:**

The infodemic created by the COVID-19 pandemic has created several societal issues, including a rise in distrust between the public and health experts, and even a refusal of some to accept vaccination; some sources suggest that 1 in 4 Americans will refuse the vaccine. This social concern can be traced to the level of digitization today, particularly in the form of social media.

**Objective:**

The goal of the research is to determine an optimal social media algorithm, one which is able to reduce the number of cases of misinformation and which also ensures that certain individual freedoms (eg, the freedom of expression) are maintained. After performing the analysis described herein, an algorithm was abstracted. The discovery of a set of abstract aspects of an optimal social media algorithm was the purpose of the study.

**Methods:**

As social media was the most significant contributing factor to the spread of misinformation, the team decided to examine infodemiology across various text-based platforms (Twitter, 4chan, Reddit, Parler, Facebook, and YouTube). This was done by using sentiment analysis to compare general posts with key terms flagged as misinformation (all of which concern COVID-19) to determine their verity. In gathering the data sets, both application programming interfaces (installed using Python’s pip) and pre-existing data compiled by standard scientific third parties were used.

**Results:**

The sentiment can be described using bimodal distributions for each platform, with a positive and negative peak, as well as a skewness. It was found that in some cases, misinforming posts can have up to 92.5% more negative sentiment skew compared to accurate posts.

**Conclusions:**

From this, the novel Plebeian Algorithm is proposed, which uses sentiment analysis and post popularity as metrics to flag a post as misinformation. This algorithm diverges from that of the status quo, as the Plebeian Algorithm uses a democratic process to detect and remove misinformation. A method was constructed in which content deemed as misinformation to be removed from the platform is determined by a randomly selected jury of anonymous users. This not only prevents these types of infodemics but also guarantees a more democratic way of using social media that is beneficial for repairing social trust and encouraging the public’s evidence-informed decision-making.

## Introduction

The internet is a powerful tool for spreading information; as such, it follows that it is equally powerful for spreading misinformation. In 2019, the number of social media users worldwide was 3.484 billion [1], with that number increasing year-by-year by an average of 9% [1,2]. With this increased use, the “power-user” or microinfluencer phenomenon has arisen, where popular social media accounts are able to reach large numbers of readers. This is increasingly important as more people begin to use social media as a source for news [3]. This news comes from a third party by a popular influencer, not posted or moderated by the social media companies themselves. Past analyses examining online misinformation often classify posts as misinformation using a “Point-And-Shoot” algorithm; this is the status quo. However, some algorithms will be better at combating misinformation than others. The Plebeian Algorithm creates criteria that social media websites should take into account when designing their algorithms to reduce misinformation. This reduction of misinformation is thought to be achieved by examining the correlation between sentiment and misinformation; it has been found that posts containing misinformation tend to have more negative sentiment when compared categorically to other posts covering the same issue [4]. Due to this correlation, it is hypothesized that an algorithm that encourages positive interactions will also reduce the amount of misinformation present on the platform through a democratic manner.

Misinformation is a key problem, yet many terms are confused in studies. Herein, the authors shall define several key terms that are often used interchangeably but whose definitions are specific and distinct. First, misinformation shall be defined as the spread, intentional or otherwise, of false information [5]. The intentions of the individuals spreading the information is irrelevant. Second, disinformation is the purposeful spread of false information [5]. A similar yet distinct definition is malinformation, which is the malicious spread of false or misleading information [5]. Finally, fake news is defined as any misinformation (with or without intention) that readers interpret as trustworthy news [5]. For this study, misinformation will be studied in-depth; however, it should be noted that future supplemental studies could conduct a similar investigation focused on disinformation, malinformation, or fake news. Infodemics can additionally be applied to the realm of health care; infodemics have the potential to intensify outbreaks when there is uncertainty among the public concerning evidence-informed preventative and protective health measures [6].

Prior to investigating the spread of misinformation, it is pertinent to define the concept of infodemiology and misinformation. This research paper defines an infodemic according to the World Health Organization (WHO) as “too much information including false or misleading information in digital and physical environments during a disease outbreak,” which “causes confusion and risk-taking behaviours that can harm health [and] also leads to mistrust in health authorities and undermines the public health response” [6]. The study of the spread of infodemics on a large scale, especially pertaining to medical misinformation, is known as infodemiology. The WHO has additionally linked the rapid surge of such infodemics during the COVID-19 pandemic to “growing digitization,” which can support the global reach of information but can also quickly amplify malicious or fabricated messages [6]. The second relevant definition is that of the concept of misinformation, which has been defined similarly to the definition used by the WHO in relation to the infodemic [6], but specifically refers to the distinct lack of verity in information related to a specific field.

At their core, most social media websites aim to maximize the amount of time that users spend on their platforms. This maximization of user page time leads to companies using highly specialized and trained machine learning to advertise content on users’ feeds [7]. At the same time, this can have unintended adverse effects such as maximizing the time a user engages with content that is not verified for accuracy. The proposed solution to this disparity between engagement and integrity is to create democratically moderated spaces. Democratic spaces and recommendations to posts with more positive sentiment are integral concepts in the Plebeian Algorithm, based on the latest evidence that misinformation tends to be more negative [5]. The Plebeian Algorithm is an algorithm, described herein, for the purpose of the control of the spread of misinformation on social media. It is beneficial compared to other existing algorithms, as will become evident. The currently implemented point-and-shoot algorithms are hypertuned to specific sources of misinformation surrounding specific topics. However, they are not adaptable to the fluidity of the definition of true information.

As mentioned earlier, most social media platforms work on a model similar to Twitter, Facebook, or YouTube where content is recommended based on user engagement [7]; however, this is not true to the same extent for all websites. One example of a website that breaks the expectations for social media algorithms is 4chan. 4chan is an excellent epitome of ephemeral social media, where content is completely anonymous and is rapidly discarded regardless of popularity [8]; in addition, there is close to no moderation and the content tends to be more negative in sentiment. This is also exemplified in Parler, an alternative social media platform established in September 2018 that aimed to bring forth a platform with total freedom of speech. Consequently, Parler attracted those who were banned from other social media websites, creating “echo chambers, harbouring dangerous conspiracies and violent extremist groups” [9] such as those who were involved in raiding the US Capitol on January 6, 2021. Reddit also has a forum-based system similar to 4chan. However, individual fora on these platforms have moderators who work to combat negative sentiment throughout the website. Reddit’s issue lies in its incredibly isolated fora, as tailoring one’s feed to be a vast majority of explicitly handpicked fora is a part of the experience; this allows for some fora to have little to no moderation [10].

There have been several related works of research in the field of misinformation detection on social media platforms. These works include studies on the connection between misinformation and cognitive psychology [11], the analysis of geospatial infodemiology [12], the effect of recommendation algorithms on infodemiology [13], the use of distributed consensus algorithms to curb the spread of misinformation [14], and the naming conventions used for viruses [15]. Although these works are in alignment with this study, they do not propose the same solution. The study that offers a solution closest to that proposed by the Plebeian Algorithm discusses the efficacy of curbing the spread of misinformation through layperson judgements [16]. Notably, this work discusses the merits surrounding a layperson algorithm but does not make suggestions for its implementation.

The objective of the study will be to determine the optimal social media algorithm to reduce the spread of misinformation while ensuring personal freedoms. The investigation conducted in this paper will have far-reaching implications that will alter how misinformation in social media platforms is addressed.

The three major implications include:

- The creation of a more open and democratic environment on social media platforms
- An overall reduction in political divisiveness and extremist sentiment both online and offline
- An increase in informed users who can make well-informed opinions on subjects

## Methods

A detailed step-based methodology was used to analyze data throughout the research process. Python 3.9 (Python Software Foundation) was the language of choice through all aspects of the project. All libraries used can be accessed using pip. The visualization of data was performed using the matplotlib and seaborn libraries in Python. Application programming interfaces (APIs) were used from Twitter, Reddit, and 4chan, gathering data regarding username, date, post, and text. Furthermore, two data sets were gathered from academic sources, containing post data from Twitter [17] and Parler [9]. Various Python libraries were used to interact and connect with the APIs, including twarc, urllib3, and basc_py4chan. The following Python libraries were used to clean the data: beautifulsoup4, demoji, and pyenchant. The pandas library for Python was used to retrieve and store third-party data sets [8,17-20], and the numpy library was used for various array operations. Finally, the nltk library was used to perform sentiment analysis, and sklearn was used to perform regressions.

Python was selected due to its ease of connectivity to the various APIs; it is well supported among a strong community, and as such, connecting to various APIs was done through prewritten libraries. This reduced the programming time while increasing the efficiency and reliability of the code.

In three of the social media services for which APIs were used (ie, Twitter, Reddit, and 4chan), four steps were performed: (1) gather data using the API and the associated Python library; (2) clean data to create a Python set of strings, containing no URLs (removed using regular expressions), HTML (removed using beautifulsoup4), usernames (removed using regular expressions), emojis (replaced with text using demoji), or non-English language (removed using pyenchant); (3) perform sentiment analysis using nltk’s SentimentIntensityAnalyzer class; and (4) save the cleaned and sentiment analyzed data frame as a pickle file. A visualization script was then programmed to display the sentiment data gathered from the social media post data. To ensure confidentiality of users, only aggregate data was displayed. Plotting a histogram with a kernel density estimation (KDE) resulted in the various graphs produced by the research team. Data for which sentiment analysis returned inconclusive due to textual limitations was removed from visualization. Limiting the language to English has the benefit of statistical comparison congruence. One notable platform that did not use an API is Facebook. The reason for this is due to the restrictions placed on the Facebook API, in terms of depth and breadth of research.

**Figure 1:**
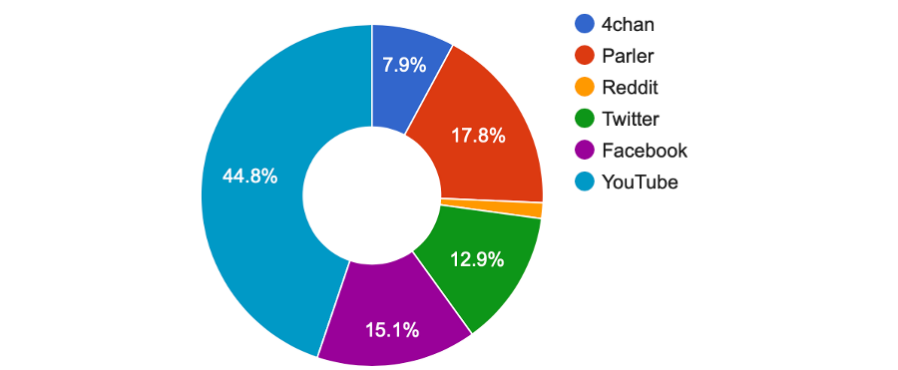
Social media data breakdown.

The six social media services analyzed (4chan, Twitter, Parler, Reddit, YouTube, and Facebook) had various amounts of associated data. A breakdown of the data analyzed herein is described in Figure 1.

The sentiment analysis dictionary selected for the analysis performed herein is the Valence Aware Dictionary and Sentiment Reasoner (VADER). This dictionary was selected as it is the industry standard for a wide array of general statement analyses and is especially recognized for producing highly accurate results with social media platforms. As such, VADER is the optimal dictionary for the purposes of this research. Although ideal algorithms should implement various checks and balances for the sentiment analysis system implemented, this paper shall focus on strictly the VADER dictionary, which is solely positive and negative sentiment. Other sentiment analysis tools exist to examine specific emotions (including anger, fear, surprise, happiness, etc).

Key term analysis was used for the data cleaning process to determine which strings were classified as relating to a specific topic. The key terms were gathered using a list of the most commonly held terms gathered from Twitter that were directly associated with misinformation.

To confirm the academic literature [5] regarding the correlation between negative sentiment and verity of information, the analysis was performed using Twitter. Data was filtered such that only Tweets containing a set of potentially misinformative keywords were assigned to be assessed using a sentiment analysis. Both were plotted through histogram, and the KDEs were compared (relative to each respective maxima).

Misinformation is directly correlated to negativity. A misinformative post is often negative in sentiment. However, this is not a certainty. As such, when determining an optimal algorithm, it will be critical to use sentiment analysis to narrow the potential misinformative candidates and then to use further methods—a jury process—to accurately detect misinformation.

The study defines several mathematical terms. Many of the histograms and KDEs as previously described form a bimodal distribution. The polarity score upon which the two peaks are centered is termed *μ*^+^ and *μ*^–^, where the sign indicates whether the term refers to the positive or negative peak. The other variable defined is the skewness of the distribution as a whole, which is described using the symbol γ. When the positive peak is the major mode, then *γ* ∈ (0, ∞). Contrarily, when the positive peak is the minor mode, then *γ* ∈ (–∞, 0). The frequency function *f* describes the frequency curve represented by the KDE (such that *f(p)* represents the frequency of strings with polarity score *p*). The skewness is calculated using the following equation:




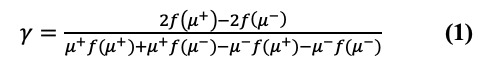




This equation for skewness was derived using the following derivation:




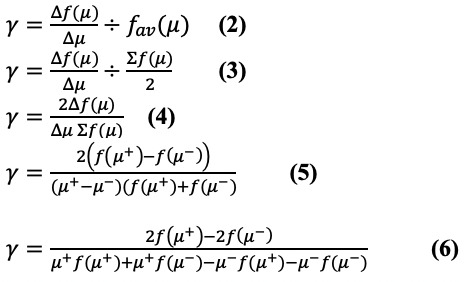




## Results

The results of the analysis will be divided by the social media platform. They will be presented in the following order:

- Reddit
- 4chan
- Facebook
- YouTube
- Parler
- Twitter

### Reddit

**Figure 2:**
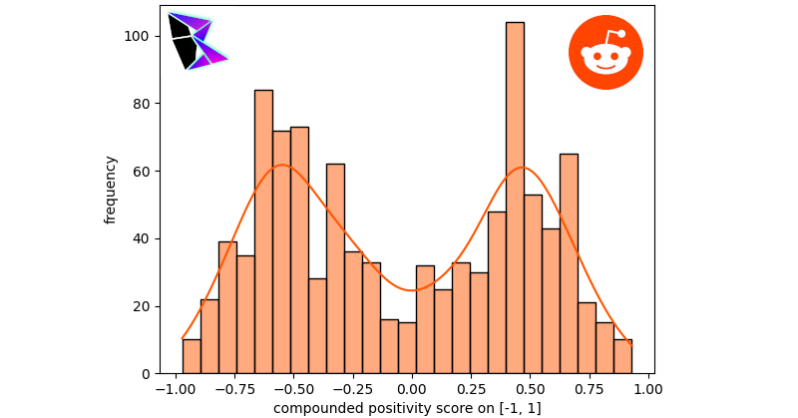
Reddit r/AskReddit frequency of positivity histogram.

When analyzing Reddit’s data, a series of subreddits were selected. The subreddits selected were r/AskReddit, r/AskThe_Donald, r/conspiracy, r/covid, r/kindness, r/movies, r/politics, and r/EnoughTrumpSpam. These subreddits were selected as an array of options, allowing an analysis of probable misinformative, probable truthful, and unknown sources. The data was gathered using the Python library urllib3. The first subreddit to be examined herein is r/AskReddit. This subreddit tends to contain a wide variety of posts from a myriad of conversation topics. As such, it is relatively indicative of Reddit overall. r/AskReddit’s histogram can be found in Figure 2. The bimodal distribution was *μ*^–^≈–0.54, *μ*^+^≈0.48, and *γ*≈–0.03214.

**Figure 3:**
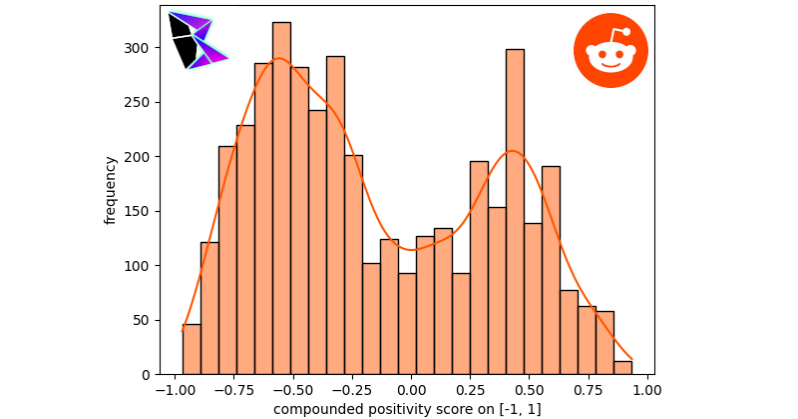
Reddit r/politics frequency of positivity histogram.

**Figure 4:**
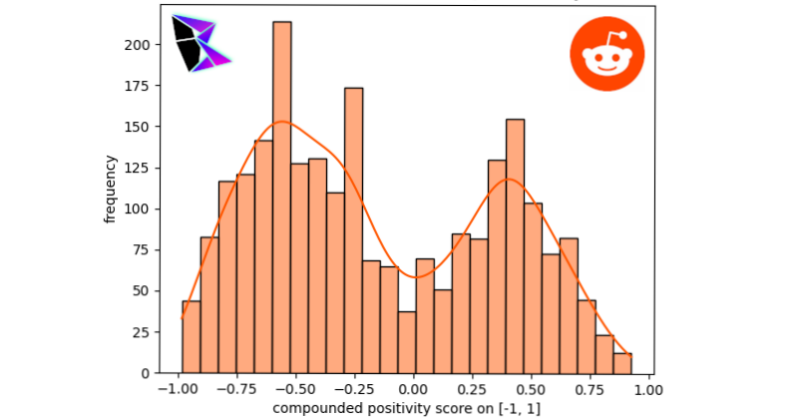
Reddit r/conspiracy frequency of positivity histogram.

It should be noted that the extremal frequencies of the bimodal distribution were approximately equal between the negative and positive peaks. Another notable subreddit examined was r/politics, which provided a sample of posts potentially swayed by the political leaning of Reddit users. The histogram and KDE for this analysis is displayed in Figure 3. The bimodal distribution for r/politics was *μ*^–^≈–0.56, *μ*^+^≈0.43, and *γ*≈–0.37776.

r/politics’s content had a stronger negative skew as is apparent by the KDE. The final subreddit to be examined is an avant-garde subreddit: r/conspiracy. In this community, users share various conspiracy theories. When one scrolls through r/conspiracy, plenty of misinformation can easily be noted, including misinformation surrounding Flat Earth Theory and QAnon. The histogram for r/conspiracy is found in Figure 4. The bimodal distribution for r/conspiracy was *μ*^–^≈–0.56, *μ*^+^≈0.39, and *γ*≈–0.33904.

As can be noted by r/conspiracy, the conspiratorial posts (which are known to contain a large volume of misinformation) are more often negative. This can be noted due to the difference in the peaks of the bimodal distribution.

### 4chan

**Figure 5:**
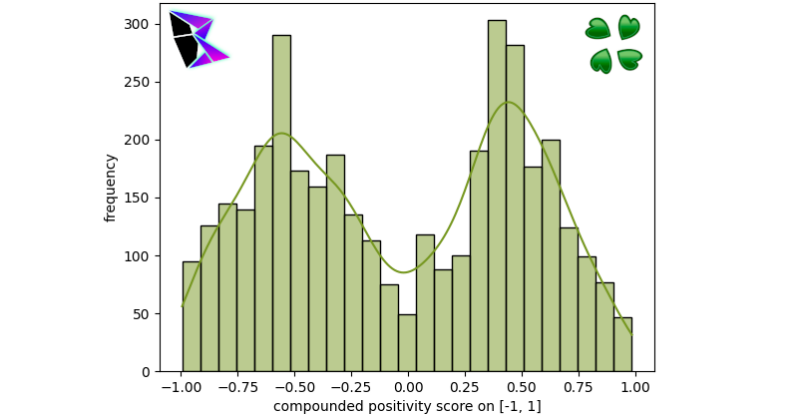
The 4chan /b/ frequency of positivity histogram.

To analyze the 4chan data, five boards were selected: /b/, /a/, /v/, /pol/, and /r9k/. These five boards were selected due to their high post frequency compared to other 4chan boards. The data was gathered using basc_py4chan. For each of these boards, a histogram was plotted (with an overlayed KDE) with 30 bins. A visualization for the histogram for /b/’s sentiment can be found in Figure 5. /b/ is described as the random board, containing a wide mixture of conversation from across 4chan. The bimodal distribution for /b/ was *μ*^–^≈–0.55, *μ*^+^≈0.46, and *γ*≈0.11380.

**Figure 6:**
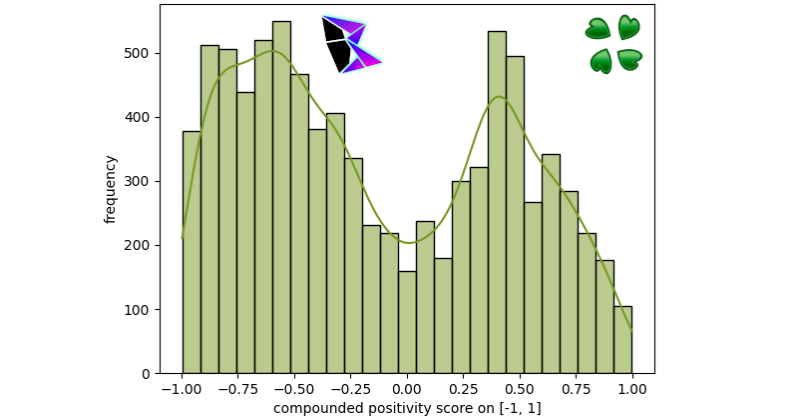
The 4chan /pol/ frequency of positivity histogram.

Another board to be visualized in this report is the visualization for the sentiment of /pol/, which can be found in Figure 6; /pol/ contains political discussion. The bimodal distribution for /pol/ was *μ*^–^≈–0.61, *μ*^+^≈0.38, and *γ*≈–0.16559.

It should be noted that the levels of extreme negative sentiment (ie, with a polarization score of less than 0.75) are substantially higher in /pol/ compared to /b/. This demonstrates that political topics tend to be more negative on 4chan.

Overall, it should be noted that 4chan consistently contains a large number of negative posts, which is greatly dependent upon the topic of the board. Boards which pertain to specific recreational activities (eg, /v/ for video games or /a/ for anime) have a lesser degree of negative polarity.

### Facebook

It is pertinent for this paper to perform an analysis on Facebook, which is currently the social platform with the largest user base of 2.8 billion active monthly users [21]. Facebook has proven to be the social media platform with the highest user base, and as such it is pertinent for this paper to perform analysis on data collected for Facebook. A data set that specifically contains data predating the COVID-19 pandemic was accessed to broaden the scope of the sentiment analysis [22]. The data set includes data gathered from Facebook’s inception until 2017. A data set with a random selection of Facebook comments from the temporal range [22] was selected for sentiment analysis using VADER.

**Figure 7:**
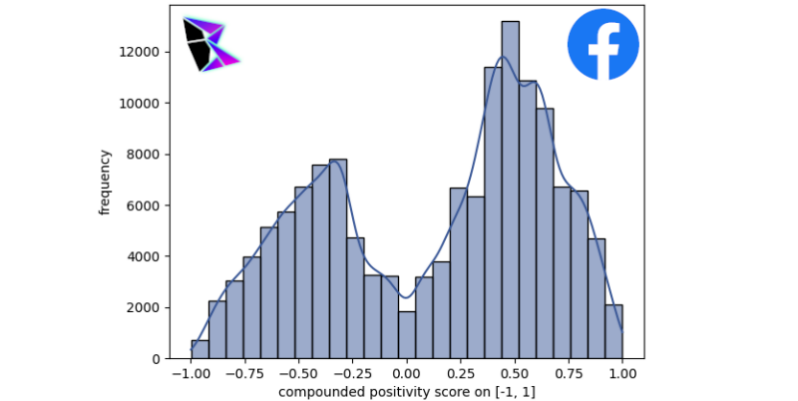
Facebook frequency of positivity histogram.

In Figure 7, a histogram was plotted with 30 bins, depicting the frequency of Facebook comments at various sentiment analytic levels. A KDE was overlayed onto the plot to show the general trend.

Notable features of the bimodal histogram include the sharp positive peak and wide negative peak. It should be noted that the integral for the KDE is as follows:




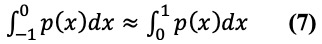




Furthermore, the following values were extracted from the KDE: *μ*^+^≈0.43, *μ*^–^≈–0.29, and *γ*≈0.49858.

### YouTube

**Figure 8:**
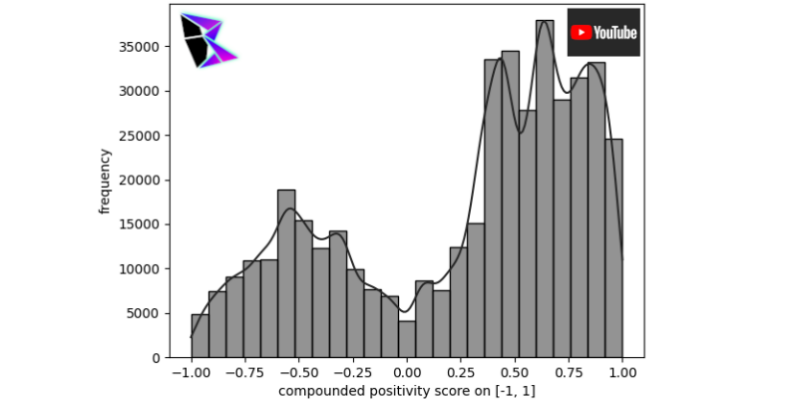
YouTube COVID-19 frequency of positivity histogram.

YouTube is based entirely on long-form video content and tends itself toward more in-depth topics. A preselected data set of YouTube comments [23], after sentiment analysis, has been visualized and presented in Figure 8.

The data set [22] was collected in 2017 and, as such, does not contain misinformation related to COVID-19. This helps to broaden the temporal scope of this analysis and ensure that the present trends hold in data outside of the COVID-19 pandemic (ie, prior to January 2020). It was also limited in geographic scope to the United States, the United Kingdom, and Canada. This limitation was due to the availability of data. It should be noted that these three countries represent the English-speaking members of the Group of Seven, a group of the seven most democratic, affluent, and pluralist nations in the world.

As can be noted, there was a strong positive skewness in the data, with *μ*^+^≈0.67, *μ*^–^≈–0.52, and *γ*≈0.66593. The high positive skewness should be noted for these YouTube comments. Potential explanations for this trend will be discussed in a later section.

### Parler

The analysis of Parler was a transition from the traditional analyses of Reddit and 4chan, due to the fact that Parler is not broken down into communities to which users subscribe but is a single news feed–style system. The analysis of Parler should be contrasted to the analysis of Twitter in the subsequent section, as users migrated from Twitter to Parler due to a perception of limitations placed on their freedom of expression on Twitter.

**Figure 9:**
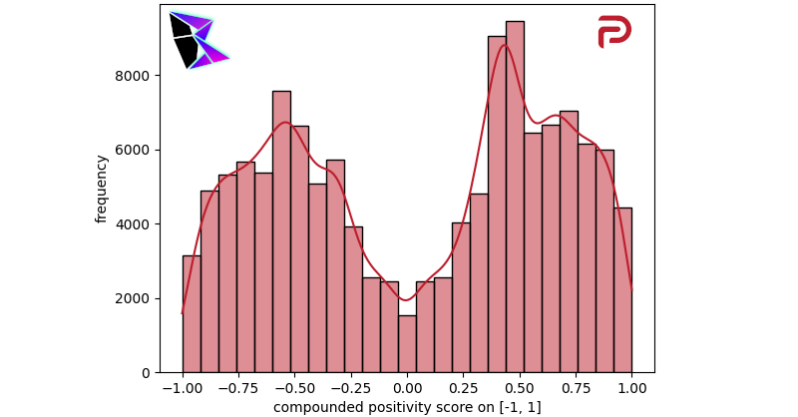
Parler COVID-19 frequency of positivity histogram.

When analyzing Parler, data was collected into a data set throughout the COVID-19 pandemic and the period surrounding the events of January 6, 2021 [9]. Figure 9 contains a visualization of the COVID-19–related parleys posted between January 2020 and March 2020. The bimodal distribution was *μ*^–^≈–0.53, *μ*^+^≈0.45, and *γ*≈0.22063.

### Twitter

The majority of the analysis performed through this paper was on the social media service Twitter. The reason for this is due to the high amount of data regarding misinformation on the platform, the overall popularity of the platform as a general case study, and the generality of the platform (compared to some other unorthodox data sources such as YouTube comments).

Similar to Parler, Twitter tweets are made at-large to the public. There are no channels, boards, or subreddits of any sort. However, due to the Twitter algorithm, there is an allowance of individuals’ feeds to be in an echo chamber. Evidently, echo chambers should be avoided wherever possible. Echo chambers are a large contributor to the rampant spread of misinformation that is seen surrounding the COVID-19 pandemic [24].

This study used a combination of both data gathered from the Twitter API and a data set of pregathered COVID-19 tweets [17,18,20]. The interface used to connect the Python code (and sentiment analysis) to the Twitter API was twarc. The reason for this duplication of analysis was to ensure that the data used was accurate. Precision must be maintained in both data collected by APIs and over a long duration.

In both studies (using the API and the data set [17]), the study analyzed the broad sentiment of COVID-19–related tweets and filtered the data by keyword. The keywords used included terms concerning misinformation surrounding COVID-19, including “China Virus,” “Bioweapon,” and “Microchip.” The filtered data then underwent sentiment analysis. Both sentimentally analyzed data were plotted on the standard histogram with overlaid KDE.

**Figure 10:**
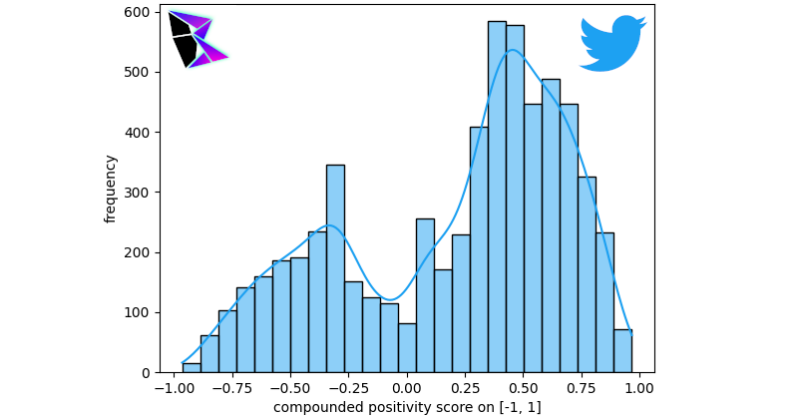
Unfiltered COVID-19 Twitter application programming interface frequency of positivity histogram.

For the discussion, the study focused on the data gathered from the Twitter API, since a similar methodology was used for gathering data for the other social media platforms studied. However, it should be noted that similar results were attained using the data set [17]. Figure 10 is a graph of the Twitter API’s gathered tweets pertaining to COVID-19 (the broad topic), where the sentiments of the tweets are plotted on a histogram with a KDE. A random sample of the data was taken for this analysis, as there were too many tweets to reasonably analyze the population. The bimodal distribution was *μ*^–^≈–0.36, *μ*^+^≈0.47, and *γ*≈0.86500.

**Figure 11:**
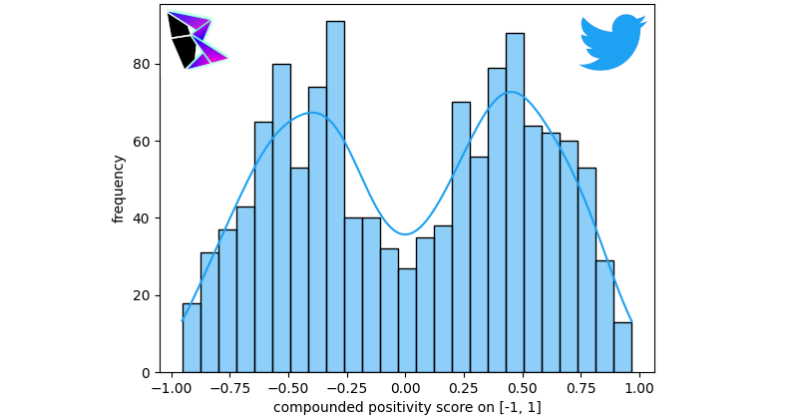
Filtered COVID-19 Twitter API frequency of positivity histogram. API: application programming interface.

As can be noted, the positive peak for the KDE is nearly double the negative peak. This indicates that the number of positive tweets far exceeds the number of negative tweets. Comparatively in Figure 11, the negative peak of the bimodal distribution is on par with the positive peak. This figure is a histographic representation of the polarity score for tweets after being filtered. The tweets selected only contain terms that are known to pertain to COVID-19 misinformation. The bimodal distribution was *μ*^–^≈–0.42, *μ*^+^≈0.47, and *γ*≈0.80080 between the two Twitter measurements.

The study’s discussion of the reasoning behind this proportional increase in the negative peak compared to the positive peak will be discussed further in the subsequent section. Again, it must be noted that the same results can be seen when performing an identical analysis on the data gathered from the data set [17].

## Discussion

In the Discussion section, not only will an analysis of the results and errors be explored but also the Plebeian Algorithm and its benefits will be discussed, as well as how it compares to the algorithms of the social media platforms studied.

### Results Analysis

Several critical notes must be made with regard to the analysis of the quantitative features produced in the Results section.

First, it is critical to note that 4chan was the only social media platform studied that had an overall positive γ, notably on the all-encompassing board of /b/. It was gathered by the observations that a system that provided users with the freedom to determine which content got promoted—as opposed to an artificial intelligence algorithm—improved the sentiment of the average post. This is a key point in the Plebeian Algorithm, which is described in a subsequent section. Second, Twitter had a more moderate skew (ie, closer to 0, or neutral) *μ*^–^, indicating that users tended to be more positive than users on the other social media platforms analyzed in the study.

It is also critical to recall that there was a strong correlation between polarity scores as determined by a sentiment analysis algorithm and the verity of the information communicated [5]. As such, the analysis provided herein can be applied to both the sentiment and the verity of a social media post.

### Echo Chambers

In analyzing the data represented by the KDEs and the skew of the bimodal distribution of the data toward negative sentiment, a confirmation of the echo chamber effect (a theory that states “the Internet has produced sets of isolated homogeneous echo chambers, where similar opinions reinforce each other and lead to attitude polarization” [25]) was clearly shown, as negative sentiment has a clear association with emotions such as anger, which have been shown to “...[reinforce] echo chamber dynamics...in the digital public sphere” [25]. In fact, other studies have also predicted the link to this effect to be the impact of the specific algorithms used in the virtual space [26]. The link gives strong evidence to suggest that the algorithms currently deployed by social media companies are creating the optimal medium through which misinformed opinions and content can grow and go uncontested. These echo chambers ensure that users are unable to get access to arguments that conflict with their beliefs and expand their perspectives.

### Sources of Error

Although the study attempted to limit error, there remained several sources of error stemming from the methodology of analysis used. The first source of error is the trouble with using key term searching, as it would give us results of posts of not only individuals spreading misinformation but also those trying to bring attention to the issue of misinformation and those who spread it. Furthermore, the sentiment analysis would also have been unable to differentiate between a misinformed post and one that tried to bring attention to the problem. This is because some of the true tweets demonstrate overall negative emotions. The final problem with the key term search is that some of the key terms determined to be misinformative may in the future be proven to be accurate information.

A further source of potential error comes in the form of the social media platforms used. One problem is that only four social media platforms were assessed, thus limiting the scope of the study. It could be that the trends found in the research will not show up in other social media platforms (eg, Facebook). Another issue from the sources assessed was that they were all only textually based and thus the method proposed may not be replicable for more graphically based social media platforms (eg, YouTube, Instagram, or Snapchat).

### Definition and Implementation of the Plebeian Algorithm

As described previously, the Plebeian Algorithm is a novel algorithm for identifying and removing misinformation through democratic means. It works in two distinct phases: the Flag Phase, to determine which posts are misinformative, and the Jury Phase, to judge the information to determine if removal is appropriate.

#### Flag Phase

The Flag Phase is tasked with the determination of possible misinformed posts. In doing so, the algorithm selects posts that have a large number of views and then performs sentiment analysis on both the original post and a “without-replacement simple random sample” [27] of comments or replies to the post. If the overall sentiment leans negative, then the post is flagged as being potentially misleading. The posts flagged will then be passed into the Jury Phase.

#### Jury Phase

The Jury Phase is tasked with the trial and removal of truly misinformative posts. These posts are removed from the home page or news feed of the user. During the Jury Phase, flagged posts are sent to a random selection of anonymous users known as jurors. This selection should provide a diverse group, consisting of varying political opinions to give the post a fair trial. The selection of jurors uses a “without-replacement simple random sample” of a population [27]. The number of jurors selected is exactly 10% of the number of viewers of a post, rounded up. It should be noted, however, that jurors are not forced to participate or vote. It is assumed that the number of voting jurors will be far less than the total number of jurors. Thus, selecting 10% of the population allows room for the uncertainty of juror engagement. The jurors are then asked to vote either for or against the removal of the post. Once the deliberation has lasted a set duration (or a threshold of response has been met), the results will be counted and the post will remain on the site or be removed by the algorithm.

**Figure 12:**
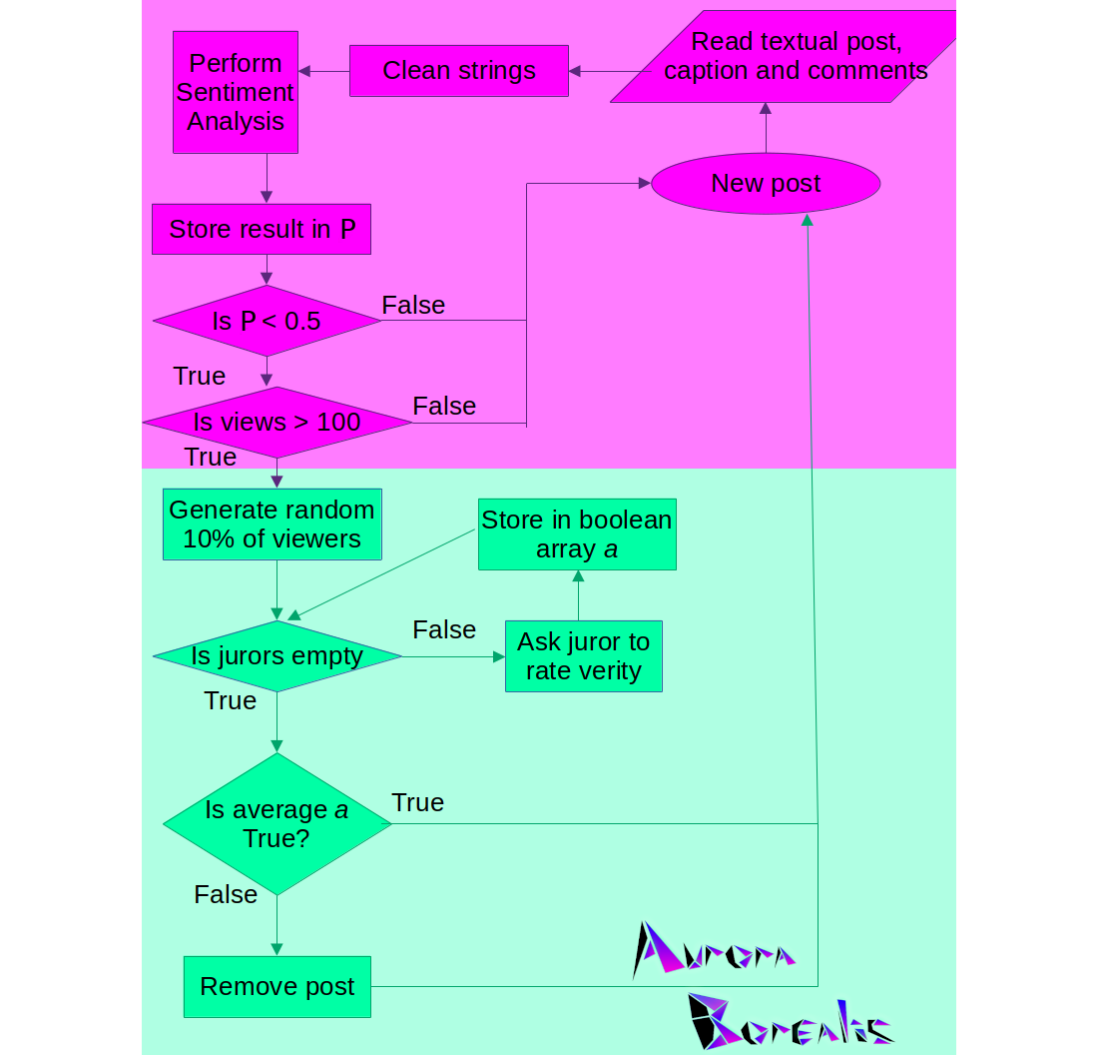
Flowchart of the Plebeian Algorithm.

For reference, a summative flowchart detailing the Plebeian Algorithm as described can be found in Figure 12. The areas colored in magenta constitute the Flag Phase, while the areas colored in mint green constitute the Jury Phase.

### Existing Algorithms

In the following section, each of the algorithms will be detailed for Reddit, 4chan, Parler, and Twitter. These analyses will be based on academic journal articles [10-12,28,29]. It is pertinent to analyze these on a case-by-case basis, as it must be ensured that the user base remains loyal to the brand and platform [30]. Prevalent existing algorithms include the PageRank and Hits algorithms [31].

Before dissecting the individual social media algorithms that are currently being used for the various platforms, it is critical to mention that most of these algorithms have an identical goal: to get users to stay on the platform, thus ensuring a continued revenue for their organization. This objective contradicts the goal of preventing misinformation from spreading on the platform, as preventing misinformation requires some censorship, resulting in a reduction of revenue. This, however, is not to indicate that the Plebeian Algorithm is of little value to site entrepreneurs. It is critical to give note that the ultimate goal of social media companies (with the exception of Parler) have already shown themselves interested in curbing and moderating their own social media platforms through their implementation of “point-and-shoot” algorithms as well as censorship of high-profile posts and accounts (eg, Twitter banning @realDonaldTrump). However, as has already been described, these algorithms are not effective at accomplishing their mission of reducing misinformation and, further, have caused users to become disillusioned with the service. This has led to many users joining platforms that capitalize on this disillusionment (eg, Parler). Hence, by implementing the Plebeian Algorithm, these social media companies finally have a method that carefully balances moderation with freedom of expression that will reinspire a sense of awe within their user base and bring back the notion of social media being a fun online space where people can collaborate and share freely.

#### Reddit

The algorithm used for Reddit is a simple upvote/downvote system, as was described in the Introduction section. Users of Reddit are encouraged to upvote content they like and are encouraged to downvote content that they do not like. Posts with more upvotes are more widely shared, whereas the opposite is true with posts with more downvotes. In Reddit, users are allowed to vote on the original post and any comments. “Comment trees” are inherently created by the system as users comment on comments (thereby chaining comments together into a treelike formation).

The Reddit algorithm is tailored to the interests of the Reddit user. Through a system of subscriptions to various topics of conversations or subreddits. Users will receive a mixture of content from the subreddits to which they have subscribed, with additional, sporadic advertisement.

The system is essentially tailored to the specific user. This contrasts with the Plebeian Algorithm, which emphasizes the democratic process for the determination of verity by the user base. Currently, Reddit contains no user-controlled means to fight misinformation aside from the “Report” button, which brings the issue to the attention of a staff person at Reddit. This process is considered a manual review by the corporation, and as such, it does not constitute something similar to the Plebeian Algorithm. For Reddit to implement a Plebeian Algorithm, it must ensure that the process of the misinformation determination remains in the hands of the user base.

Although Reddit currently appears to be a democratic system, it is more of a fiefdom [29]. For example, in 2013 the r/FindBostonBombers subreddit slandered the Brown family by connecting them with the 2013 attack on the Boston Marathon at the direction of the moderators of the subreddit [32]. Examples like this resonate throughout Reddit through incidents such as “the Fappening,” where nude photographs were released to the public unbeknown to victims. Incidents like these make apparent the crux of the fundamental issue with Reddit: the moderators. This promotes content moderation by a few elite members of communities, instead of by the members of the said community as a whole.

It should be noted that Reddit is a platform that is built on a sense of anonymity. Users are not required to add their personal email addresses or their real names. As is the case in all the implementations of the Plebeian Algorithm, it is important that the social media company critically analyzes the existing market served and existing qualities that users may be drawn to. An implementation of the Plebeian Algorithm on Reddit should preserve user anonymity and should still not require the use of personal emails or real full names.

#### 4chan

The 4chan algorithm is similar to that of Reddit; it uses a system whereby the audience determines whether or not content is viewable to its users. In contrast to Reddit, 4chan uses an ephemeral system for its content [10]. 4chan is also divided into several boards that encapsulate distinct topics of conversation. Furthermore, any content can be posted on the /b/ board, as this board’s topic is described as “Random” [10]. A second critical aspect of the 4chan algorithm is the notion of anonymity. 4chan encourages its user base to remain anonymous through their posts. Over 90% of posts and comments on /b/, the most popular board on 4chan, are anonymous [10].

4chan is the algorithm that is nearest to the proposed Plebeian Algorithm; however, there are subtle yet notable differences. The Plebeian Algorithm does not incorporate any notions of anonymity nor ephemerality. Content must be both traceable and permanently recorded. This will help assure that the goals of the social media companies at-large (which often differ from the goals of 4chan) remain consistent. Keeping the goals consistent for each individual social media platform will be essential to ensure that the users of the platform remain loyal while gaining the added benefits that the Plebeian Algorithm offers.

For 4chan’s algorithm to become a Plebeian Algorithm, it should remove its ephemerality. This would be essential to ensure that content has the time to undergo the process. Content posted on 4chan’s /b/ often lasts less than 1 minute [10]. As such, the Plebeian Algorithm would not have the time to undergo both two phases (the Flag and Jury Phases), a critical step that is necessary to the algorithm’s democratic approach.

#### Facebook

Facebook is a valuable selection, demonstrating a powerful social media platform and a tailored user experience; its popularity makes it useful for analysis. Although the Facebook company is not wholly transparent [9], the company has announced it highly favors personalized content of users (eg, posts from close friends and private groups) to that of public groups and pages to which the user likes and follows [33,34]. This presented a limitation for the analysis of Facebook data, as ideally, data had to be collected through preselected data sets for quantitative analysis [24].

There are many differences between the Facebook algorithm and the Plebeian Algorithm. The method used by Facebook, particularly during the COVID-19 pandemic, aims to combat the spread of misinformation and is based on neural networks trained to search for key terms in textual elements (including posts, comments, and statuses). It should also be noted that Facebook’s algorithm includes a large amount of human work, which is easily biased. As has been stated in prior sections, there are several issues with this method of misinformation censorship; most notably, the Facebook algorithm is limited in scope to a specific subset of misinformation topics. Algorithms of this nature will detect specific key terms such as “COVID” included in the text of a post to provide additional information and resources for viewers; these algorithms will also provide information to the user sharing the post before publicizing the post. On the contrary, the Plebeian Algorithm is proactive in nature: it is universally applicable to all forms of misinformation and works to combat infodemics before they become widespread. As stated previously, infodemics of misinformation have led to the problem of pandemics becoming exacerbated and thus harder to control for public health workers. By implementing the Plebeian Algorithm, public health will be improved, especially concerning future pandemics, as potentially dangerous misrepresentations or falsehoods about the situation will be contained to a smaller percentage of the populace, and thus ensures that reliable and trustworthy information is more accessible and widespread. The Plebeian Algorithm also requires less maintenance by developers, actively running automatically without the requirement of hard coding key terms to flag.

For Facebook to implement a Plebeian Algorithm, a high degree of planning would be required. Since Facebook is the most prevalent social media platform, a gradual implementation based on a rolling basis is recommended. AB testing should be used to ensure a smooth and successful implementation. Facebook should automate and democratize their home page algorithm to implement a Plebeian Algorithm for its service.

#### YouTube

Due to the inherent difficulty in performing visual sentiment analysis for videos, comments of YouTube videos were analyzed. This does not give a complete picture of the YouTube algorithm, which attempts to keep users engaged longer on the website by presenting a tailored feed; the end goal being that the algorithm can predict videos the user would like to watch before they search [35]. This algorithm looks at a range of user data including watch time, closing a video tab, the user’s interests, freshness, and user interactions with the video [35]. This algorithm has proven highly effective at finding and distributing viral content.

By aligning with the user’s sentiment, the algorithm can effectively produce more positive comments, as seen in Figure 8. A sentiment filter used by YouTube includes the removal of videos that do not meet advertiser guidelines [36]. The main difference between YouTube’s current content moderation approach and a Plebeian Algorithm’s implementation of content moderation is the democratic aspect of content removal. This is made clear with many of YouTube’s controversies within their community revolving around a lack of communication and censorship of larger creators [37].

The moderation system of YouTube is already a form of the Plebeian Algorithm with users being able to like and dislike comments or videos, in addition to reporting them if they are unwanted. The main disconnect between this and the Plebeian Algorithm is that, when a comment or video is reported, there is no public jury phase where the community decides if it stays. This has become clear with YouTube’s controversies within the community revolving around issues such as the lack of communication and censorship of YouTube influencer Logan Paul. Should YouTube implement the Plebeian Algorithm, a Jury Phase is required after content is reported and prior to its removal process. It should also be noted that YouTube’s jurors are not a random distribution. The moderation algorithms are programmed by humans, and as such, it is extremely difficult to ensure that the correct decisions are consistently being made. Artificial intelligence forms the basis of the YouTube algorithm, but the plebeian jury is replaced with a judge, who may be easily persuaded or hold personal biases. The wisdom of the crowd phenomenon (quod vide) plays a substantial role in the use of the jury for the Plebeian Algorithm.

#### Parler

Parler uses a more typical algorithm. It limits posts to 1000 characters and circulates them to the user base at-large. Thus, unlike Reddit and 4chan, there are no communities in which content is posted on Parler. Parler was founded as a promoter of the freedom of speech, and as such, its user base is highly concerned with a lack of censorship on their posts [11].

Although this may at face value appear to be in direct opposition to the implementation of any algorithm, it is important to note that the Plebeian Algorithm ensures that any and all decisions regarding the verity of information remain in the users’ hands. Parler would still benefit from implementing a Plebeian Algorithm, as it would preserve Parler’s ultimate goal (to promote freedom of expression) while limiting the spread of misinformation.

For Parler to implement a Plebeian Algorithm, it must implement both the Flag and Jury Phases of the Plebeian Algorithm. Notably, the preservation of the freedom of expression on the platform must be ensured above all. This will ensure that the user base remains loyal and supportive of the change and does not boycott Parler or switch to a new social media platform (as they have already migrated from Twitter). The Parler user base is notably precarious, and it must ensure that the user base remains loyal to the platform. This should be done through proper marketing of the transition, which is to be discussed later.

#### Twitter

Finally, Twitter uses a similar algorithm (in opposition still to Reddit and 4chan) in that posts and content are released to the user base at-large. The techniques of this algorithm particularly means that misinformation is more likely to spread on Twitter (and Parler) compared to other platforms (eg, Reddit and 4chan). The large user base on Twitter and the widespread availability of data must be taken into account, as it will be crucial that the culture and atmosphere of Twitter are maintained to ensure that the user base remains content with any algorithmic changes. Twitter’s executives most likely would be interested in increasing their reach by attempting to regain the trust of those who migrated to Parler. These individuals are highly concerned with a decrease in censorship and an increase in the freedom of expression. They believe that the social media platform should remain separate from the process of promotion and demotion of content [11].

For Twitter to implement a Plebeian Algorithm, it must attempt to promote the freedom of expression and a decrease in censorship while also maintaining their reliability. This is done using the Plebeian Algorithm, which takes advantage of both concerns. Layperson algorithms have proven effective at curbing the spread of misinformation and at increasing reliability [18]. According to the definition by Epstein et al [16], the Plebeian Algorithm would be classified as a type of layperson algorithm. Twitter would need to place a level of trust in the layperson to provide the user base with liberty while maintaining truth in the content posted.

### Condorcet’s Jury Theorem

As was researched in the 18th century by the Marquis de Condorcet, the Condorcet’s jury theorem [38] clearly justifies the need for the Jury Phase in the Plebeian Algorithm. The theorem describes the behavior of a larger number of individuals selected to sit on a jury to judge the crimes of another individual. Proposed by Condorcet (and later proven by numerous mathematicians and statisticians in the late 20th century), the theorem explains that two scenarios may unfold when attempting to determine the truth by means of polling a sample of the population [38]. First, if the sample’s understanding of the topic is poor, their judgement will not be certain. In this situation, the optimal sample size would be a single individual, as increasing the number of jurors will only increase the uncertainty [38].

However, a jury implemented in the Plebeian Algorithm should take Condorcet’s jury theorem into account, by ensuring that the jury falls into the second scenario. The second type of jury would occur when the jury’s knowledge of the subject is relatively high or is perceived as relatively high [38]. As such, an optimal Plebeian model would be passive, instead of aggressive, in its UI/UX. It should be ensured that acting as a juror is entirely optional and is opt-in instead of opt-out. The user interface should be minimal to ensure that the public reception of the implementation of the Plebeian Algorithm is positive. Although this will likely decrease the percentage of the sample who opt-in to act as jurors, consistence will be achieved due to the positive reception of the algorithm implementation. An ideal Plebeian Algorithm implementation to secure the second subset of juried defined by Condorcet’s jury theorem may go unnoticed for the average user.

Assuming that an implementation of the Plebeian Algorithm can secure its jury into the latter jury type, it would secure the wisdom of the crowd. Increasing the sample selected as potential jurors will increase the certainty. This phenomenon has been described as “wisdom of the crowd” [39]. As the sample size increases, the certainty of the decision that the jury comes to also increases. Thus, taking a sample size of 1% has a higher possibility of accidentally selecting a group of the most extreme individuals, compared to randomly selecting a sample size of 10%.

### Eradication Versus Containment

One of the benefits of the Plebeian Algorithm in comparison to the status quo is the difference between eradication and containment. The algorithms of the current system tend to use an eradication approach. They view the issue of the spread of misinformation with a narrow perspective [35], and as such, they tend to implement a “Point-And-Shoot” algorithm. With this system, media companies determine which posts contain misinformation and eradicate them on a case-by-case basis. For example, many social media companies use a COVID-19 key term search and flag posts that contain them. They then link to a government website with information on the pandemic to the post.

This is in stark contrast to the Plebeian Algorithm, which takes a containment-based approach to the spread of misinformation. It should be noted that the technology to remove all instances of misinformation does not exist [40]. Instead, it is important that the algorithm detects as many cases of misinformation as possible and brings the rest to the broad public. This essentially “pops” any filter bubbles and echo chambers [26]. It allows for positive discussion from the community, which tends to lead toward a decrease in misinformation [4].

### Reduced Censorship

Another massive benefit of the Plebeian Algorithm is the reduction of censorship. With regard to the COVID-19 pandemic, a majority of misinformed posts has been spread by those with politically right ideologies, or Republicans. A total 48% of US Republicans believe that SARS-CoV-2 is no more dangerous than the common influenza [41] (compared to 25% of US Democrats [41]), and 42% of Republicans believe that hydroxychloroquine—a treatment for malaria—is an effective treatment for SARS-CoV-2 [41] (compared to 5% of Democrats [41]). Furthermore, Republicans (or those with political right ideologies) tend to be more concerned with the preservation of the freedoms of speech and expression. Thus, it is evident that we must preserve these freedoms for any algorithmic change to be effective. The Plebeian Algorithm goes further than this: it works to increase the rights of the individuals with respect to cross-region community matching freedom of expression. Individuals have the right to post and speak as they please and promote the spread of the information they deem to be pertinent. Additionally, they have the right to decide what content they want to see on the platform and what they do not. These benefits will help to ensure that the public reacts in a positive light to the change. Implementing a Plebeian Algorithm is a net positive; it is a positive change for both the containment of infodemics and the promotion of freedom of expression among social media users. Furthermore, under the Plebeian Algorithm, social media companies are still permitted to analyze user activity according to their privacy policies to provide appropriate advertisements tailored to the user. This will ensure that the revenues for social media companies will not be reduced in the process.

### Viral Naming Conventions

One subtopic explored herein is viral naming conventions and the connection between the name used to describe COVID-19 in relation to the level of verity in social media posts. For the purpose of this analysis, only posts on COVID-19 were considered; thus, social media platforms for which the data used herein was collected before 2020 were not analyzed (ie, Facebook and YouTube). Parler was examined at length since it uses a relatively standard social media algorithm, comparable to that of Twitter. A pickle file of Parler data, filtered to COVID-19, was generated from the Parler data set [9]. Additional filters were applied to the pickle file, as are described later.

To simplify the analysis, three categories of parleys were created on which analysis was performed separately. First, all posts mentioning COVID-19 using any naming conventions were gathered. The posts were collected using no additional filters, labelled “None.” Second, from the COVID-19 parleys, a filter was applied to gather all parleys containing viral names referring to locations including, but not limited to, “Wuhan Virus,” “China Virus,” and “Indian Variant.” All these terms have been described by the US Centers for Disease Control and Prevention (CDC) to potentially propagate misinformation and xenophobia [42,43]. This filter was termed “Locational Taxonomy” [44]. The final filter, “Biological Taxonomy” [44], refers to the biological names for COVID-19, or officially approved names by the WHO, including, but not limited to, “SARS-CoV-2,” “Alpha Variant,” and “B.1.617.” This nomenclature is used and promoted by the CDC [38] to limit xenophobia. As such, it was hypothesized that parleys using these terms would be less likely to be misinformative and more likely to have a positive sentiment [45].

**Figure 13:**
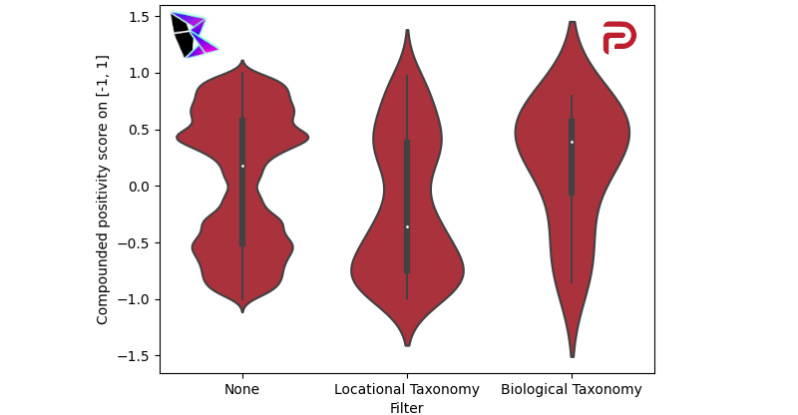
Viral naming conventions on Parler: violin plot.

Sentiment analysis was performed on all three filtered data sets, and the results were plotted as a violin plot in Figure 13. Each subplot portrays the three filters as discrete categories along the x-axis (ie, “None,” “Locational Taxonomy,” and “Biological Taxonomy”) against the compounded polarity score on [–1, 1]. Each subplot visualizes a KDE that is rotated vertically for ease of visualization and a pictorial representation of the median, mean, first quartile, and third quartile.

This visualization provides exceptionally relevant results. The data filtered to COVID-19 at-large was similar to the KDEs plotted for all of the social media algorithms discussed in the Results section, demonstrating a precise bimodal distribution with a positive and negative peak, and a neutral trough. The violin plots for the locational and biological taxonomies verified the hypothesis. The locational taxonomy filter showed strong negative sentiment, implying a higher likelihood of misinformation. In contrast, the biological taxonomy filter showed a strong positive sentiment, implying a greater degree of verity.

It is important to note that the findings are not limited to COVID-19. Similar findings were discovered (not pertaining to social media) relating to the 2009 H1N1/09 pandemic [46,47] and the 1918 Spanish Flu pandemic [15], among others [48,49], for which there were concerns surrounding xenophobic viral nomenclature. It is also pertinent to discuss the specific limitation surrounding the use of COVID-19 data for this analysis. It is often difficult for populations to alter their vocabulary to change the reference of a xenophobic initial name to an accurate descriptor [50]. Specifically, with regard to COVID-19 variants of concern (VoCs), many scientific sources still note the location of the VoC’s discovery. This brings two significant points to the forefront of discussion: first, national health agencies need to provide precise and nonxenophobic nomenclature from the onset of pandemics, and second, the use of locational taxonomy should not automatically flag a post (ie, it should be flagged through sentimental analysis solely). The Plebeian Algorithm assists in this analysis, as it does not consider specific search terms, but rather pure sentiment. This handles issues surrounding truthful posts containing locational taxonomy. The lack of consensus among the scientific community should be noted regarding the potential benefits and drawbacks of using locational taxonomy [15,45].

### Geolocation

There exists a critical connection between the virtual and physical worlds as it pertains to the spread of misinformation and various consequences therefrom. Several studies have been conducted hereupon. One fundamental limitation posed by the use of social media platforms to track the spread of misinformation is the inability to deal with the spread of misinformation in more personal settings (eg, face-to-face interactions, videoconferencing, and direct messaging). Thus, a thorough study of the translation of misinformation from social media platforms to real-world phenomena will be conducted.

Myriad studies have been conducted surrounding infodemics [51-53]. This includes the correlation between geolocation of social media connections and various social determinants (eg, race, sex, and socioeconomic status) [51], and a study [54] determining optimal methods of geolocation on social media. Two additional studies discussed the sociological consequences of geolocation in the context of social media, namely, the detection and reduction of youth cannabis consumption [52] and the applications of geolocation to urban planning [53].

Furthermore, studies suggest that there exists a strong correlation between trends on social media and events such as COVID-19 [12,51]. Evidently, any change on social media will have real-world impacts. Thus, it is apparent that a reduction in the amount of misinforming content in a social media user’s home page corresponds with a reduction in the likelihood that they will propagate misinformative statements when having in-person conversations. Successful implementation of the Plebeian Algorithm will limit the spread of misinformation on social media platforms and in the lives of their users.

### Public Reaction

Skeptics of the Plebeian Algorithm might be concerned that such a massive alteration of the social media algorithm will incite hesitancy from the public. Whether this hesitance takes the form of negative feedback or boycotting, it is extremely legitimate and must be dealt with. Many will point to the 4chan platform as a negative example of an algorithm that offers user discretion regarding the promotion of content instead of a corporate algorithm.

#### Marketing

This paper will first argue that the major difference between the two strategies lies within the realm of marketing. Marketing is a critical aspect of any social media company, especially when undergoing massive changes. In fact, some broad-scale social changes require marketing strategies [55]. Companies must ensure that the Plebeian Algorithm is adapted to meet the specific needs and goals of the social media company and its user base. For this reason, the Plebeian Algorithm is simply a suggested implementation, with a footnote that the algorithm must be highly adapted to the unique situation. Every social media company has varying objectives, such as Facebook’s aim to connect friends, Reddit’s goal to create conversations between like-minded individuals, and Parler’s goal of preserving freedom of expression.

An effective marketing strategy for the transition to the Plebeian Algorithm ensures that users are aware that the overall atmosphere of the social media platform will not be altered. Promotion of the current atmosphere must take priority, lest the change face backlash by users. There is a potential, should improper marketing be implemented, that overly moderated individuals may leave the social media platform, leaving those with more extreme (and often misinformed) views to take over the widespread content of the platform. However, adequate marketing that emphasizes the static nature of the culture and social atmosphere of the platform during the transition alleviates this concern.

#### Feedback of Current Algorithms

Second, this paper will discuss the feedback on the current algorithms as provided by the community. This feedback consists of discussions on social media platforms about each platform’s algorithm. An analysis of preselected opinion pieces was performed [33,34,56-62]. These opinion pieces were sourced from well-known news or magazine sources, discussing the various social media platforms analyzed herein.

Overall, there is substantial desire for social media platforms to be more democratic in their algorithm. It is also widely believed among many social media users that, to improve algorithms, companies should implement a more transparent algorithm. Currently, algorithms vary widely and the functionality of most are not publicly available information. Changes improving transparency tend toward positive user feedback on the platform.

It is also critical to note that, for any implementation of the Plebeian Algorithm, a post must exceed a popularity threshold to be flagged in the Flag Phase. It is essential for the social media platforms to adapt their current algorithm to the determination of this popularity threshold. The goal of most current algorithms is to show users popular content that they may enjoy based on past interests. This can be done through a plethora of metrics, including likes, views, comments, recency of the post (termed “freshness”) [35], and more. For example, the Twitter algorithm tends to prioritize the number of comments, whereas the YouTube algorithm prioritizes freshness.

### Implementation

Another concern of a potential implementing platform of the Plebeian Algorithm would be the technological requirements of the implementation, including storage and processing power required to conduct the Plebeian Algorithm on their millions of posts. Furthermore, this application of the Plebeian Algorithm would need to be a continuous process, ensuring that the algorithm continually updates when new comments are added to a post. As has been shown herein, the inclusion of comments increases the level of detail. All the data analyses visualized herein included comments and the text of the original post. As such, the computational power required appears to be great. There are, however, many alterations that can be made to the Plebeian Algorithm to reduce computation costs.

First, the Plebeian Algorithm does not need to be updated with the post of every new comment. It can be performed on intervals, whereby a subsection of posts is checked for new comments at every time interval. These new comments (and only the new comments) are then sent through sentiment analysis. In terms of data storage, it may prove useful for the social media platform to store a single additional byte of data for each post. The bit of highest significance, referred to as the “Flag of Need Determination,” represented as *ϕ_d_* can be defined using the following equation:




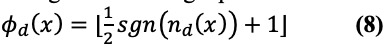




such that:




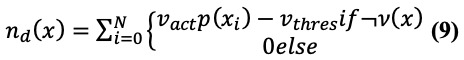




where *n_d_* represents the value of determination (which is not scaled), *x* represents a thread, *x_i_* represents a specific comment or post within a thread, *ν* is a Boolean function returning a high value if the comment is new and low if it has been analyzed, sgn represents the signum function, *v_act_* and *v_thres_* represent the actual and threshold popularity of a thread in number of views, and *N* represents the number of posts or comments in the thread.

If high, the post or thread can be safely skipped by the algorithm. If low, the post or thread will be analyzed to ensure that no misinformation goes undetected. The remaining seven bits of the data represent the sentiment of the entire thread, represented using *β_N_*, where *N* is the number of comments in the post, excluding the original post. These bits can be calculated using the following equations:




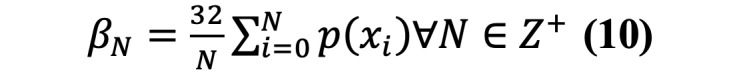




In some circumstances, it may be more computationally convenient to calculate *β_N_* recursively, which may be done using the following:




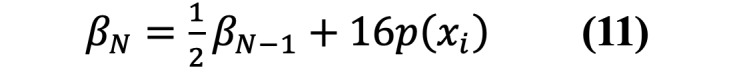




These equations demonstrate that a byte can be associated with each thread to decrease the processing requirements to execute the Plebeian Algorithm on a large scale.

It should also be noted that the Plebeian Algorithm is a machine learning model. It can be built to work in tandem with existing machine learning algorithms, thus decreasing the computing power required. Data storage is minimized using the one-byte storage method previously described. As is the case with all neural networks, the Plebeian Algorithm’s Flag Phase will increase in accuracy over time by manipulating the string data as a validation set. Thus, the neural network will improve in accuracy over time. Due to time and resource limitations, the paper used the VADER; however, to increase Flag Phase precision over time, it is recommended that platforms implement the VADER sentiment analysis tool initially but build on it to adapt to the specific lexicon of the social media platform at the period of time. This accounts for minor differences in various social media algorithms and for lexical changes over time.

It is critical that a public release of the Plebeian Algorithm should be done through a process of AB testing. To efficiently fix any inevitable bugs present in the implementation of the algorithm (including any potential philosophical issues surrounding a specific realization/implementation), AB testing will be vital in the assurance that users consuming media under the new algorithm remain loyal to the brand and minimize any potential negative impacts. It will allow user feedback to be gathered for the small subsection of users presented with the Plebeian Algorithm implementation.

### Limitations

Although the Plebeian Algorithm is a great replacement for the current attempts by social media platforms to reduce the spread of misinformation, it is limited by several key factors. First, as stated earlier, the algorithm was only confirmed applicable for strictly text-based social media platforms and posts. Thus, the moderation of videos or images are outside of the scope of its use. Second, private sources of media such as chat rooms and servers are not within the scope of the algorithm, and thus, the algorithm is limited to public communication media. Third, the determination of a popularity threshold can be problematic. On Twitter, for example, a significant number of retweets are done passively (ie, they are not done for the express purpose of sharing with others but are done subconsciously by the user). Passive sharing may cause issues in the determination of whether a piece of content meets a popularity threshold. Finally, it is limited in the sense that it cannot determine what is misinformation at an instantaneous time selection, and as such, misinformation cannot be extracted from the algorithm at any time.

### Conclusions

The implications of this research are significant as to provide social media platforms with a new flagging method that uses sentiment analysis. This will be critical in the detection and prevention of infodemics and using a democratic approach that gives the power to the social media user to ultimately decide what content should be on the platform based on accuracy. The Plebeian Algorithm directly reduces political polarization and extremist ideas, which create a divide among users and improve cooperation on resolving key issues and problems plaguing humanity and restoring the trust between the public and experts.

Additionally, it is predicted that this will result in more reliable social media platforms, leading to an overall reduction of ignorance and misinformed opinions among users. Finally, the model created will lead to users expressing themselves without concern of the political viewpoint of the social media platform. Inherently, this also minimizes the impact of external biases, such as political climate, as those who vote will be completely random and anonymous.

Many areas of research remained unanalyzed. These topics include, but are not limited to:

- Conducting a study on the use of the Plebeian Algorithm on a selection of social media platforms and detecting the amount of misinformation over time after its implementation (ie, a real-world tested example) that would then be compared to current methods used, such as the aforementioned “Point-And-Shoot” Algorithm
- Creating a type of sentiment analysis for graphical content that could examine the emotion within an image to determine if it could be misinformation (eg, Snapchat, Instagram, and TikTok) [63,64]
- Determining the spread of misinformation correlated with the spread of viruses—this could be useful in predetermining locations (and users by extension) who are at higher risk of being exposed to or expounding misinformation
- Exploring the applicability of the Plebeian Algorithm in surveillance contexts, including for criminal investigations, employee onboarding, and health care [65-68]
- Analyzing the spread of misinformation through online vendors such as Amazon or eBay. In particular, recent audits of Amazon (as of 2021) show a dangerous disregard for reliable information, for example, presenting vaccine misinformation books along with well-cited vaccine information books in generic searches for vaccine information [69-73]
- Applying models of higher sophistication for data analysis and visualization (which requires access to more in-depth data), including term frequency–inverse document measures [74] and Levenshtein distances [75] among others [76]
- Examining the optimal method of implementation and integration for the Plebeian Algorithm with various existing networking systems and infrastructures
- Continuing analysis of data collected to corroborate to prior studies on behavioral impacts of the sentiment of informative posts on social media
- Analyzing the role of corporate social media platforms (ie, Slack) in the dissemination of misinformation, especially in private chat channels
- Examining the misinformation containment models using juries, including the jury system implemented by Wikipedia
- Analyzing the rise of audio-form content, including podcasts, Clubhouse, and Spotify Greenroom audio-chat rooms, for the potential spread of misinformation—many of these media are becoming increasingly influential sources of news and information for many [77]
- Exploring the connection between location-based social media apps (eg, Foursquare) at the spread of geographic misinformation [78]

COVID-19 has had substantial impacts upon modern society. Optimists hoped these impacts would prove to unite a polarized world in the spirit of cooperation and global security. Although this has happened, their hopeful unity to the political schism has not. The Plebeian Algorithm is not a vaccine for an infodemic; however, it is a treatment to help curb and prevent the virus of misinformation from continuing to spread and grow out of control. This has the critical side-effect of putting power back in the hands of the people and removing the potential domination of a single entity (eg, a social media company) who may be swayed by external forces when deciding if content should be removed. All in all, it is recommended that social media executives consider the implementation of a variation of the Plebeian Algorithm, explicitly modified to adapt to the specifics of the platform. This will help curb misinformation both with regard to the COVID-19 infodemic and to prevent future infodemics.

## Abbreviations

API: application programming interface
CDC: Centers for Disease Control and Prevention
KDE: kernel density estimation
VADER: Valence Aware Dictionary and Sentiment Reasoner
VoC: variant of concern
WHO: World Health Organization
